# Complete genome sequence of *Propionibacterium freudenreichii* DSM 20271^T^

**DOI:** 10.1186/s40793-015-0082-1

**Published:** 2015-10-24

**Authors:** Patrik Koskinen, Paulina Deptula, Olli-Pekka Smolander, Fitsum Tamene, Juhana Kammonen, Kirsi Savijoki, Lars Paulin, Vieno Piironen, Petri Auvinen, Pekka Varmanen

**Affiliations:** Institute of Biotechnology, University of Helsinki, PO Box 56 (Viikinkaari 9), 00014 Helsinki, Finland; Department of Food and Environmental Sciences, University of Helsinki, PO Box 66 (Agnes Sjöbergin katu 2), 00014 Helsinki, Finland

**Keywords:** +*Propionibacterium*, Type strain, Dairy starter, B12 vitamin

## Abstract

*Propionibacterium freudenreichii* subsp. *freudenreichii* DSM 20271^T^ is the type strain of species *Propionibacterium freudenreichii* that has a long history of safe use in the production dairy products and B12 vitamin. *P. freudenreichii* is the type species of the genus *Propionibacterium* which contains Gram-positive, non-motile and non-sporeforming bacteria with a high G + C content. We describe the genome of *P. freudenreichii* subsp. *freudenreichii* DSM 20271^T^ consisting of a 2,649,166 bp chromosome containing 2320 protein-coding genes and 50 RNA-only encoding genes.

## Introduction

Strain DSM 20271^T^ (= van Niel 1928^T^ = ATCC 6207) is the type strain of species *Propionibacterium freudenreichii*, which is the type species of its genus *Propionibacterium* [1]. There are traditionally two groups described in *Propionibacterium* genus; the “classical” or “dairy” and the “cutaneous” propionibacteria. *P. freudenreichii* belongs to the dairy group and is divided into two subspecies on the basis of lactose fermentation and nitrate reductase activity. The DSM 20271^T^ strain represents the *P. freudenreichii* subsp. *freudenreichii* distinguished from subsp. *shermanii* by nitrate reduction and by a lack of lactose fermentation. [1]. Dairy propionibacteria do not belong to human microbiota but can be isolated from various habitats including raw milk, dairy products, soil and fermenting food and plant materials such as silage and fermenting olives [1]. Strains of *P. freudenreichii* have a long history of safe use in human diet and for instance in the production of Swiss-type cheeses, in which they play central role as ripening starters [1, 2]. Industrial applications of *P. freudenreichii* include production of vitamin B12 (cobalamin), as well as several other biomolecules like propionic acid, trehalose and conjugated linoleic acid [3]. Recently, there has been growing interest to study *P. freudenreichii* for its probiotic properties. Complete genome sequence of the type strain *P. freudenreichii* subsp. *shermanii* CIRM-BIA1 has been reported [4], but lack of other complete genome sequences has prevented the genomic level comparisons between the two subspecies. Thus, the genomic analysis of DSM 20271^T^ strain should help us in *P. freudenrichii* subspecies definition that has been under debate [4, 5].

Here we present a summary classification and a set of features for *P. freudenreichii* DSM 20271^T^, together with the description of the complete genomic sequence and annotation.

## Organism information

### Classification and features

**Fig. 1:**
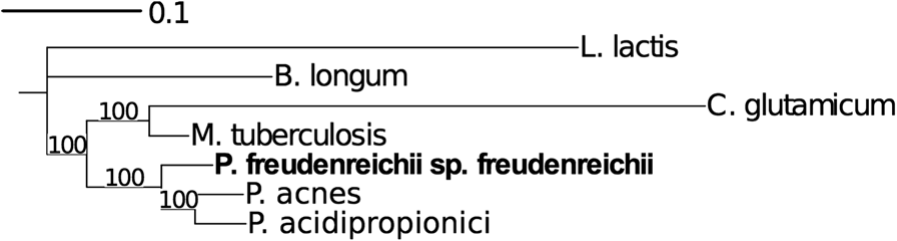
Phylogenetic tree showing the relationship of *Propionibacterium freudenreichii* ssp. *freudenreichii* DSM 20271^T^ (shown in bold print) to *Mycobacterium tuberculosis*, *Corynebacterium glutamicum*, *Bifidobacterium longum*, *Propionibacterium acnes*, *Propionibacterium acidipropionici* and *Lactococcus lactis* (outgroup). Tree is based on MAFFT [21] aligned 16 s rRNA gene. The tree was built using the Maximum-Likelihood method [22] with GAMMA model. Bootstrap analysis with 500 replicates was performed to assess the support of the tree topology. Tree was visualized with iTOL [23]

**Fig. 2:**
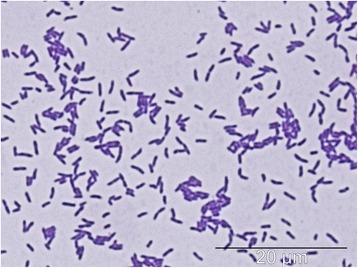
Optical microscope image. The cells of strain 20271 grown for 72 h, Gram stained. Image from light microspope, magnification 100x

*P. freudenreichii* subsp. *freudenreichii* DSM 20271 T is a Gram-positive, non-motile, non-sporulating, anaerobic to aerotolerant, mesophilic *Actinobacteria* belonging to the order *Propionibacterium*. The strain was originally isolated as one of the three propionic acid-producing strains from Emmental cheese by von Freudenreich and Orla-Jensen as Bacterium acidi propionici a [6] during their work in Bern, Switzerland [7]. The strain was further studied by van Niel and renamed to *Propionibacterium freudenreichii* [6]. Figure 1 shows the phylogenetic neighborhood of DSM 20271^T^ in a 16S rRNA sequence based tree. Cells of DSM 20271^T^ are short rods with length of approximately 1,5 *μ*m (Fig. 2). According to API 50 CH (Biomerieux, France) carbohydrate fermentation test the growth of DSM 20271^T^ is supported by carbon sources including glucose, fructose, mannose, glycerol, adonitol, inositol, erythritol and galactose (Table 1).

**Table 1:** Classification and general features of *Propionibacterium freudenreichii* subspecies *freudenreichii* DSM20271 T according to the MIGS recommendations [24]

MIGS ID	Property	Term	Evidence code^a^
	Classification	Domain Bacteria	TAS [25]
		Phylum *Actinobacteria*	TAS [26, 27]
		Class *Actinobacteria*	TAS [26, 27]
		Order *Propionbacteriales*	TAS [28]
		Family *Propionibacteriaceae*	TAS [1]
		Genus *Propionibacterium*	TAS [1, 29]
		Species *Propionibacterium freudenreichii* subspecies *freudenreichii*	TAS [1, 29, 30]
		(Type) strain: van Niel 1928 T, (DSM 20271 T = ATCC 6207)	
	Gram stain	*Positive*	TAS [1]
	Cell shape	*Rod*	TAS [1]
	Motility	*Non-motile*	TAS [1]
	Sporulation	*Not reported*	NAS
	Temperature range	*Mesophile*	TAS [1]
	Optimum temperature	*30 °C*	TAS [1]
	pH range; Optimum	*~4.5–8; ~7*	NAS
	Carbon source	*Glucose, fructose, mannose, glycerol, adonitol, inositol, erythritol, galactose*	IDA
MIGS-6	Habitat	*Swiss cheese*	TAS []
MIGS-6.3	Salinity	*Unknown*	TAS []
MIGS-22	Oxygen requirement	*Anaerobic*	TAS [1]
MIGS-15	Biotic relationship	*Free-living*	NAS
MIGS-14	Pathogenicity	*Non-pathogen*	NAS
MIGS-4	Geographic location	*Unknown*	NAS
MIGS-5	Sample collection	*Unknown*	NAS
MIGS-4.1	Latitude	*Unknown*	NAS
MIGS-4.2	Longitude	*Unknown*	NAS
MIGS-4.4	Altitude	*Unknown*	NAS

^a^Evidence codes - *IDA* inferred from direct assay, *TAS* traceable author statement (i.e., a direct report exists in the literature), *NAS* non-traceable author statement (i.e., not directly observed for the living, isolated sample, but based on a generally accepted property for the species, or anecdotal evidence). These evidence codes are from the Gene Ontology project [31]

**Table 2:** Genome sequencing project information for *Propionibacterium freudenreichii* DSM 20271^T^

MIGS ID	Property	Term
MIGS 31	Finishing quality	Finished
MIGS-28	Libraries used	One PacBio 10 kb standard library
MIGS 29	Sequencing platforms	PacBio RS II
MIGS 31.2	Fold coverage	198x
MIGS 30	Assemblers	SMRTAnalysis (2.1.0), HGAP2
MIGS 32	Gene calling method	Prodigal v2.50
	Locus Tag	RM25
	Genbank ID	CP010341
	GenBank Date of Release	February 1^st^ 2015
	GOLD ID	Gs0113908
	BIOPROJECT	PRJNA269789
MIGS 13	Source Material Identifier	DSM 20271^T^
	Project relevance	Type strain, dairy starter, B12 vitamin

## Genome sequencing information

### Genome project

This organism was selected for sequencing on the basis of its importance in food fermentations and in metabolite production.

### Growth conditions and genomic DNA preparation

The strain was grown to early stationary growth phase in propionic medium (PPA), composed of 5.0 g. tryptone (Sigma-Aldrich), 10.0 g. yeast extract (Becton, Dickinson), 14.0 ml 60 % w/w DL-sodium lactate (Sigma-Aldrich) per liter and pH adjusted to 6.7. The cells were harvested by centrifugation for 5 min at 21,000 g at 4 °C and washed once with 0.1 M Tris–HCl pH 8.0. The DNA extraction was performed with ILLUSTRA™ bacteria genomicPrep Mini Spin Kit (GE Healthcare) according to the manufacturer’s instruction for Gram-positive bacteria using 100 mg/ml of lysozyme (Sigma-Aldrich) and 30 min incubation time in the lysis step.

### Genome sequencing and assembly

The complete finished genome sequence of *P. freudenreichii* strain DSM 20271^T^ was generated at the Institute of Biotechnology, University of Helsinki, using Pacific Biosciences RS II sequencing platform [8](Table 2) . One standard PacBio 10 kb library was constructed and sequenced using two SMRTCells with 180 min runtime on the RS II instrument, which generated 145,463 reads totaling up to 608.98 Mbp. For the assembly, the data was filtered using default HGAP parameters. The resulting 130,046 reads totaling up to 557.87 Mbp were used to generate the initial genome sequence. 498.74 Mbp of the filtered data mapped to the assembled genome afterwards. The assembled genome sequence was generated using SMRTAnalysis (2.1.0) HGAP2 pipeline [9] with default parameters, excluding the expected genome size and seed cutoff which were set to 2,700,000 and 7000 respectively. The assembly contains one contig which represents the whole genome. The resulting assembly was further improved by two consecutive rounds of mapping the full data on the reference and obtaining a new improved consensus sequence on each run. This was done using the standard SMRTAnalysis resequencing protocol with Quiver algorithm [9]. The circular nature of the final consensus sequence was then confirmed and the start of the sequence manually set to *dnaA* using Gap4 tool from Staden package [10].

### Genome annotation

Genes were identified using Prodigal v2.50 tool [11] with manual curation in ARGO Genome Browser [12]. The predicted genes were translated and functionally annotated with description lines, Gene Ontology (GO) classes and Enzyme Commission (EC) numbers with PANNZER program [13] using UniProtKB, Enzyme and GOA databases. PfamA domains were identified using InterProScan 48.0 [14], transmembrane helices and signal peptides were found with TMHMM [15] and SignalP [16], respectively. Clusters of Orthologous Groups (COG) assignments were done by using CD-Search [17]. The tRNAscanSE tool [18] was used to identify tRNA genes and ribosomal RNA were predicted with RNAmmer v1.2 [19].

## Genome properties

**Fig. 3:**
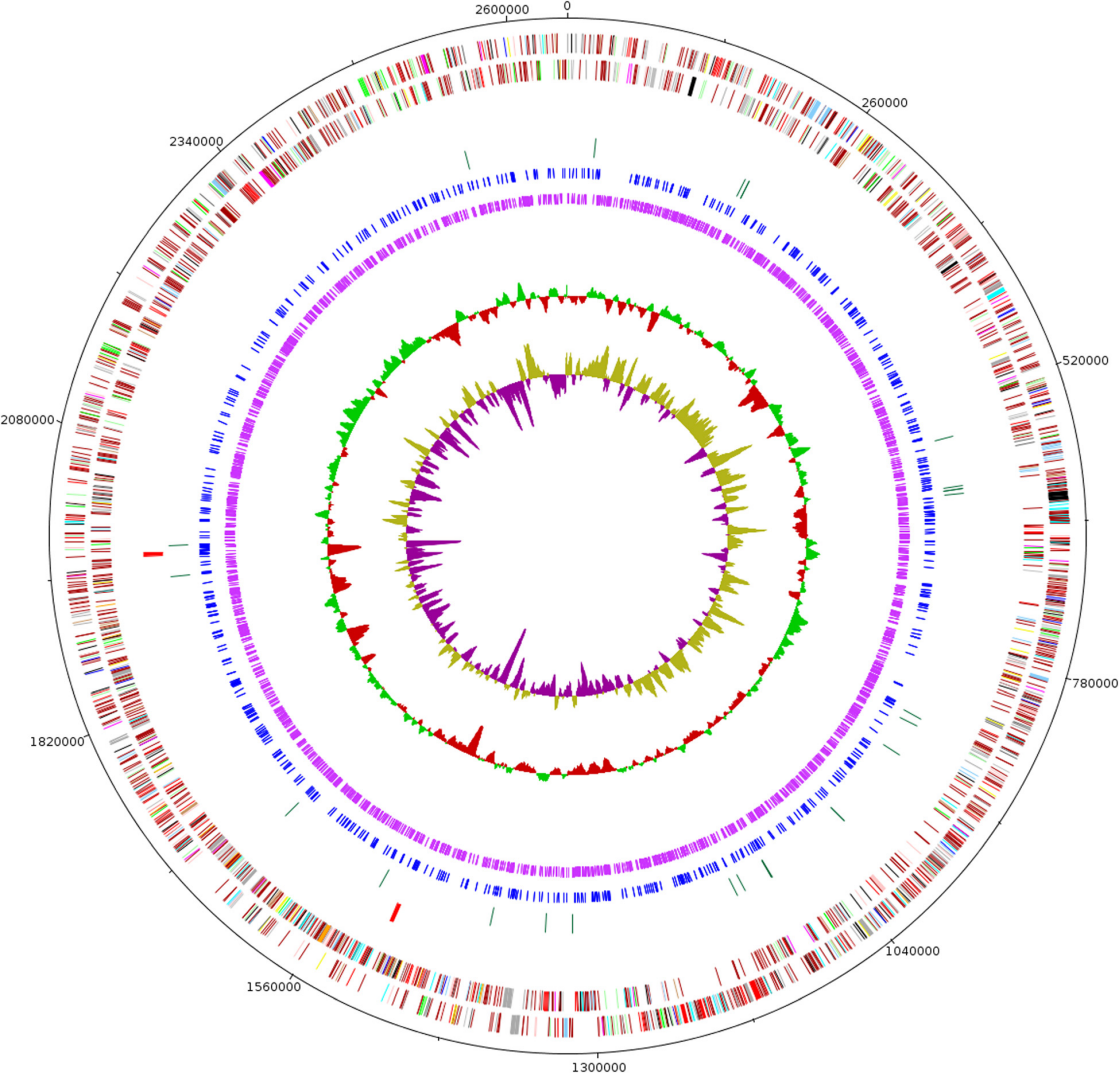
Genome properties of *Propionibacterium freudenreichii* ssp. *freudenreichii* DSM 20271^T^ . The circles are from inner to outer order: GC skew, GC%, putative ^m4^C, ^m6^A, tRNA, rRNA, CDS reverse, CDS forward. CDS are colored according to the COG functional categories

The circular genome of *Propionibacterium freudenreichii* subsp. *freudenreichii* DSM 20271^T^ is 2,649,166 nucleotides with 67.34 % GC content (Table 3) and contains one finalized chromosome with no plasmids. From total number of 2370 genes 2320 (97.9 %) are protein coding and 50 (2.1 %) are RNA genes (Fig. 3). 91.80 % of all proteins were functionally annotated whilst the remaining genes were annotated as “functionally unknown putative proteins”. The distribution of genes into COGs functional categories is presented in Table 4. Three sequence motifs containing methylated bases were also detected in the genome by PacBio sequencing and SMRTAnalysis Modification and Motif detection protocol. Two of these motifs, 5′-GG**A**NNNNNNNCTT-3′ and 5′A**A**GNNNNNNNTCC-3′, are partner motifs and correspond to same modifications on different strands with ^m6^A as a modified base on 3rd and 2nd position respectively. The modified base is shown in bold. Each motif is found 664 times in the genome and the marked bases are methylated in all of the 1328 motifs. The structure of the motifs and similarity to existing methyltransferases in REBASE [20] suggests that this is a Type I restriction-modification (RM) system. The third modified motif detected in the genome is 5′-TCGW**C**GA-3′ which partners with itself and is found 4258 times in the genome. In 3676 of these motifs (86.3 %) the 5th nucleotide (C) is found to be modified. The type of this modification could not be reliably identified. However, 319 of the detected modifications are identified as ^m4^C although with low confidence. This finding is supported by the similarity of the recognition site to existing methyltransferases found in REBASE [20] and suggests that there is a Type II RM system acting on this motif in the genome. Therefore also the unidentified modifications are probably ^m4^C bases. This data together suggest that there are two active RM systems present in *P. freudenreichii*. Comprehensive analysis of these RM systems and corresponding methylations requires further study.

**Table 3:** Genome statistics

Attribute	Value	% of Total
Genome size (bp)	2,649,166	100.00
DNA coding (bp)	2,321,778	87.64
DNA G + C (bp)	1,783,838	67.34
DNA scaffolds	1	100.00
Total genes	2353	100.00
Protein coding genes	2320	97.90
RNA genes	50	2.10
Pseudo genes	NA	NA
Genes in internal clusters	NA	NA
Genes with function prediction	2160	91.80
Genes assigned to COGs	1751	74.42
Genes with Pfam domains	1958	83.21
Genes with signal peptides	113	4.80
Genes with transmembrane helices	577	24.52
CRISPR repeats	0	0

**Table 4:** Number of genes associated with general COG functional categories

Code	Value	%age	Description
J	138	5.86	Translation, ribosomal structure and biogenesis
A	0	0.00	RNA processing and modification
K	152	6.46	Transcription
L	211	8.97	Replication, recombination and repair
B	0	0.00	Chromatin structure and dynamics
D	18	0.76	Cell cycle control, Cell division, chromosome partitioning
V	32	1.36	Defense mechanisms
T	82	3.48	Signal transduction mechanisms
M	77	3.27	Cell wall/membrane biogenesis
N	1	0.04	Cell motility
U	24	1.02	Intracellular trafficking and secretion
O	73	3.10	Posttranslational modification, protein turnover, chaperones
C	138	5.86	Energy production and conversion
G	157	6.67	Carbohydrate transport and metabolism
E	205	8.71	Amino acid transport and metabolism
F	57	2.42	Nucleotide transport and metabolism
H	106	4.50	Coenzyme transport and metabolism
I	58	2.46	Lipid transport and metabolism
P	123	5.23	Inorganic ion transport and metabolism
Q	31	1.32	Secondary metabolites biosynthesis, transport and catabolism
R	219	9.31	General function prediction only
S	80	3.40	Function unknown
-	1017	43.22	Not in COGs

The total is based on the total number of protein coding genes in the genome

### Conclusions

Prior to this report only a single genome sequence was available for *Propionibacterium freudenreichii*, from the type strain of *P. freudenreichii subsp. shermanii* CIRM-BIA1 [4]. In the present study the first genome sequence of a *P. freudenreichii subsp. freudenreichii* strain was described. *P. freudenreichii* is an industrially important species and a rare producer of biologically active form of vitamin B12. Probably the characteristics of *P. freudenreichii* DNA such as high G + C content and regions of repeated sequences have hampered the unraveling the complete genomes of this species. The results presented here indicate that PacBio RS II sequencing platform is well-suited to overcome these potential obstacles. In this study three DNA sequence motifs containing methylated bases were detected. Our future investigations include using this platform for sequencing of several additional strains for establishing core and pan-genomes as well as methylomes to gain understanding of genome structure and evolution of *P. freudenreichii*.

## Abbreviations

PPA: Propionic medium
HGAP: Hierarchical genome-assembly process
RM: Restriction-modification
